# Mechanistic insights into daidzin from *Glycine max* against breast cancer via network pharmacology and multi-level molecular modeling

**DOI:** 10.1371/journal.pone.0355672

**Published:** 2026-08-11

**Authors:** Lan Thi Vu, Luong Trong Vu, Lien Thi Kim Vu, Hang Thi Thuy Pho, Quan Huu Nguyen, Lan Thi Ngoc Nguyen, Yen Thi Hai Nguyen, Hung Duc Nguyen, Mau Hoang Chu

**Affiliations:** 1 Department of Biology, Thai Nguyen University of Sciences, Thai Nguyen, Vietnam; 2 Faculty of Biology, Thai Nguyen University of Education, Thai Nguyen, Vietnam; 3 Institute of Theoretical and Applied Research, Duy Tan University, Ha Noi, Vietnam; 4 Faculty of Natural Sciences, Duy Tan University, Da Nang, Vietnam; 5 Department of Biology, Thai Nguyen University of Medicine and Pharmacy, Thai Nguyen, Vietnam; 6 Faculty of Fundamental Sciences and Basic Medical Sciences, Pham Ngoc Thach University of Medicine, Ho Chi Minh City, Vietnam; Guru Nanak College, INDIA

## Abstract

Breast cancer remains a major cause of morbidity and mortality in women, with around 2.3 million new cases and 670,000 deaths worldwide in 2022. Daidzin, a soy isoflavone glycoside from *Glycine max*, is a candidate bioactive scaffold, but its breast cancer-relevant mechanisms remain poorly defined. This study used an integrated *in silico* strategy combining network pharmacology and molecular modeling to prioritize daidzin targets and validate key interactions, with sirtinol as a reference compound. Target prediction identified 101 putative daidzin targets, and intersection with breast cancer-associated genes yielded 97 common targets. Protein-protein interaction analysis highlighted hub genes including ALB, TNF, MMP9, CASP3, SRC, ITGB1, MMP2, ESR1, IL2, and HSP90AA1. Enrichment analyses suggested convergence on extracellular/vesicle-related functions, metallopeptidase activity, and pathway modules spanning metabolism, inflammation, endocrine signaling, and cancer circuitry. Docking against ten hub proteins produced binding energies from −6.00 to −11.49 kcal/mol, with the strongest affinity for MMP9 (6ESM; −11.49 kcal/mol), exceeding B9Z (−10.54 kcal/mol) and sirtinol (−10.59 kcal/mol). Molecular dynamics simulations indicated stable complexes, and Molecular Mechanics Generalized Born Surface Area (MMGBSA) supported stronger binding for daidzin-MMP9 (−46.86 ± 3.83 kcal/mol) than sirtinol-MMP9 (−14.12 ± 8.99 kcal/mol). Absorption, Distribution, Metabolism, Excretion, and Toxicity (ADMET) prediction indicated favorable safety-related flags for daidzin, although lower predicted intestinal absorption and Caco2 permeability than sirtinol suggest potential exposure-related limitations. Density Functional Theory (DFT) analysis supported comparatively greater electronic stability. Collectively, the results prioritize a daidzin-MMP9 axis for experimental validation.

## Introduction

Breast cancer remains the leading malignancy among women worldwide and constitutes a significant cause of cancer mortality. In 2022, an estimated 2.3 million new cases were diagnosed globally, with approximately 670,000 deaths, underscoring the sustained public health burden despite progress in screening and treatment strategies [1]. Contemporary management relies on multimodal regimens that include surgery, radiotherapy, cytotoxic chemotherapy, endocrine therapy, molecularly targeted agents, and immunotherapy, selected according to tumor stage and molecular subtype. Although these approaches have improved survival in many settings, clinical benefit is frequently constrained by treatment-limiting toxicities, cumulative organ injury, and long-term adverse events that affect adherence and quality of life [2]. In addition, therapeutic escape remains a central challenge, particularly in hormone receptor-positive disease, where endocrine resistance can emerge through rewiring of receptor signaling, compensatory growth factor pathways, and microenvironmental influences, ultimately limiting durable disease control [3].

Given these limitations, bioactive natural products and diet-derived phytochemicals are increasingly investigated as sources of chemically diverse scaffolds and as mechanistic probes for multi-pathway intervention. Soy isoflavones have drawn sustained attention because of their structural similarity to endogenous estrogens and their potential to modulate endocrine signaling with context-dependent biological outcomes [4,5]. Epidemiological syntheses have reported inverse associations between soy isoflavone consumption and breast cancer risk in several cohorts, while also emphasizing that observed effects may vary with population characteristics and exposure patterns [6,7]. Within this chemical class, daidzin, the glycosylated form of daidzein, is of interest because intestinal or microbial metabolism can influence conversion to active aglycone forms, thereby shaping systemic exposure and downstream cellular responses [8]. In addition, daidzin has been characterized as a potent and selective ALDH2 inhibitor involved in acetaldehyde detoxification, and given alcohol-linked genotoxicity and ALDH2 rs671 associations with breast cancer risk in Asian women, its targets merit a network-level study [9,10]. However, the molecular mechanisms by which daidzin mediates its biological effects remain incompletely defined.

Breast cancer progression is driven not only by tumor-intrinsic proliferation programs, but also by extracellular matrix remodeling, inflammatory signaling, and endocrine regulation. Matrix metalloproteinases such as MMP2 and MMP9 are repeatedly implicated in invasion and metastatic dissemination, and systematic evidence supports an association between elevated tumoral MMP2, MMP9 and poorer patient outcomes [11–13]. In parallel, tumor necrosis factor pathway activity contributes to tumor-microenvironment crosstalk and immune regulation; TNF receptor 2 has been reviewed as a relevant signaling axis in the breast tumor microenvironment, with links to immune evasion and disease progression [14]. These interconnected processes intersect with estrogen receptor signaling networks that are central to therapy response and endocrine resistance, reinforcing the value of mechanistic frameworks that capture pathway interdependence [15].

Network pharmacology provides a systems-level strategy to address biological complexity by integrating compound-target prediction with disease-associated gene networks and network topology. This approach facilitates prioritizing biologically interconnected targets and signaling pathways rather than relying on isolated single-protein hypotheses [16]. Moreover, integrating network pharmacology with structure-based modeling establishes an efficient framework for mechanism-oriented lead discovery by linking multi-target system-level inference with atomistic evaluation of ligand-protein interactions [17].

Previous studies have reported that extracts from germinating seeds of transgenic soybean lines contain various bioactive compounds, including flavonoids, alkaloids, phenolics, and saponins [18]. Subsequent analyses of isoflavones revealed that the total isoflavone content in germinated transgenic soybean seeds ranged from 60.60 to 63.10 mg/100 g dry weight (DW). This content comprised daidzin (15.60–18.50 mg/100 g DW), genistin (24.40–26.40 mg/100 g DW), daidzein (2.79–4.84 mg/100 g DW), genistein (2.44–5.23 mg/100 g DW), puerarin (1.15–1.48 mg/100 g DW), and glycitein (1.37–1.89 mg/100 g DW). Based on these findings, the present study focuses on daidzin as a representative bioactive compound.

Within this framework, network analysis was applied to prioritize breast cancer-related targets and pathways associated with daidzin. Subsequently, molecular docking and molecular dynamics simulations, complemented by binding free energy estimation, were employed to comparatively evaluate binding feasibility and complex stability, thereby refining potential candidate targets for further validation [19].

Accordingly, this study adopted an integrated computational strategy to elucidate the potential therapeutic relevance of daidzin in breast cancer by combining network pharmacology-based target identification and pathway enrichment with molecular docking and molecular dynamics simulations for structural validation. Furthermore, post-simulation analyses, including MMGBSA calculations, *in silico* ADMET profiling, and conceptual DFT descriptors, were incorporated to provide a multi-scale assessment of target engagement plausibility, binding energetics, and physicochemical properties. This comprehensive framework establishes a rational basis for subsequent experimental validation.

## Materials and methods

### Structure preparation of daidzin

**Fig 1:**
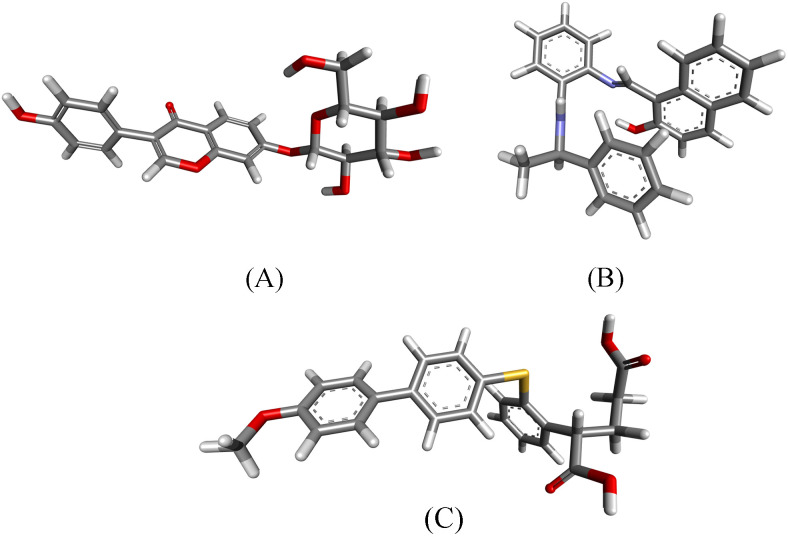
Chemical structure of daidzin (A), sirtinol (B), and B9Z (C).

Daidzin has a molecular formula C_21_H_20_O_9_ with a molecular weight of 416.1107 Da. Sirtinol (molecular formula of C_26_H_22_N_2_O_2_ and molecular weight of 394.4654 Da) was selected as a general breast cancer-related comparator, whereas B9Z, possessing a molecular formula of C_24_H_22_O_5_S and a molecular weight of 422.1188 Da, was used as the reference inhibitor bound to the 6ESM structure (Fig 1).

### Predicting targets of daidzin

Potential target information for daidzin was obtained using SwissTargetPrediction (http://www.swisstargetprediction.ch/), with access recorded on November 6, 2025. The canonical SMILES was entered into the platform, and “*Homo sapiens*” was selected as the specified species.

### Exploring targets of daidzin action on breast cancer

Breast cancer-related target datasets were collected from GeneCards (https://www.genecards.org/) and Open Targets (https://platform.opentargets.org/), with access recorded on November 6, 2025. Records from both resources were merged into a unified list, and duplicate entries were removed. Standard target nomenclature and corresponding common names were subsequently obtained from UniProtKB (https://www.uniprot.org/), visited on November 6, 2025.

### Screening common targets of daidzin and breast cancer

Common targets between daidzin and breast cancer were identified by comparative analysis using the VennDiagram package in the R environment [20]. Standardized target names were obtained from a protein knowledgebase to maintain consistency in nomenclature.

### Prediction of protein-protein interaction network

Protein-protein interaction (PPI) prediction is fundamental for delineating cellular network architecture and clarifying disease-associated mechanisms. By integrating deep learning with structure-based modeling, PPI inference can also support drug discovery by nominating therapeutic compounds and prioritizing targets, including proteins traditionally considered difficult to modulate pharmacologically. In this analysis, the interaction network associated with daidzin was examined using the STRING database. The overlapping targets identified from the Venn-based comparison were submitted to STRING, and the Homo sapiens dataset was selected to restrict inference to human-relevant interactions. The resulting protein-protein interaction network was visualized in Cytoscape version 3.10.3 [21,22]. Hub nodes and highly connected genes were ranked using the CytoHubba plugin, whereas NetworkAnalyzer was used to characterize global network features. Genes with the most significant number of connections were emphasized to indicate strong network associations. Core targets were further screened using established topological metrics, including Degree, Average Shortest Path Length, Closeness Centrality, and Clustering Coefficient, to support prioritization within the interaction network [21–23].

### Gene Ontology (GO) and Kyoto Encyclopedia of Genes and Genomes (KEGG) pathway analysis

Gene Ontology and Kyoto Encyclopedia of Genes and Genomes enrichment analyses are widely used bioinformatics strategies for interpreting large-scale gene lists, including those derived from RNA sequencing and proteomics, by identifying statistically overrepresented functions and pathways. Gene Ontology annotations organize gene products into Biological Process, Molecular Function, and Cellular Component domains [24,25]. In contrast, KEGG curation links genes to pathway-based interaction networks and provides contextual information related to diseases and pharmacological interventions [26]. GO enrichment analysis of the common targets was conducted using the DAVID database (https://davidbioinformatics.nih.gov/). Enriched terms were classified into Biological Process, Molecular Function, and Cellular Component, with statistical significance defined as p < 0.05. KEGG pathway enrichment was performed in DAVID using the same parameter settings. The 30 highest-ranked pathways were retained for downstream interpretation, and the enrichment results were presented using bar and lollipop visualizations.

### Molecular docking

Three-dimensional structures of daidzin were generated in PDB format using Biovia Discovery Studio Visualizer. The B9Z structure was obtained from the co-crystallized ligand in the MMP9 structure 6ESM and used as the target-relevant reference ligand for MMP9 docking. Polar hydrogens were added, and Gasteiger charges were computed, while all torsional bonds were defined as rotatable. Crystal structures for the top ten targets derived from the protein-protein interaction network were obtained from the RCSB Protein Data Bank (https://www.rcsb.org/). Molecular docking was conducted in AutoDock Tools. The docking grid was defined with dimensions of 60 × 60 × 60 points and a grid spacing of 0.375 Å. Target-specific grid center coordinates were used for all receptors and are provided in S1 Table. These grid centers were placed within the ligand-binding or active-site regions of the corresponding proteins to maintain reproducible docking conditions across the analyzed targets. Before docking, co-crystallized ligands were removed from the receptor structures prior to docking of daidzin and comparator ligands. Crystallographic water molecules were removed during receptor preparation. Metal ions with catalytic or structural relevance were retained. For MMP9/6ESM, the catalytic Zn² ⁺ ion was retained to preserve the metalloproteinase active-site environment, whereas B9Z was removed before docking and used as the native ligand for comparison with daidzin. Protein structures were prepared by adding polar hydrogens and assigning Kollman charges. Ligand protonation states were prepared according to the dominant forms expected under physiological pH conditions. The Lamarckian genetic algorithm was used to search for low-energy binding conformations and to prioritize energetically favorable interaction patterns. After docking, the pose with the most favorable binding energy reported in the docking output was selected for visualization in Discovery Studio Client 2024. Sirtinol was retained as a general breast cancer-related comparator rather than an MMP9-specific positive control. For MMP9 docking, B9Z, the co-crystallized inhibitor in the 6ESM structure, was included as a target-relevant reference ligand to compare daidzin binding within the same structural pocket. Redocking validation of B9Z into its native binding site of MMP9 (PDB ID: 6ESM), which would involve calculating the heavy-atom RMSD between the redocked and crystallographic poses to confirm docking grid reliability, was not performed in this study. B9Z was used solely as a target-relevant reference ligand for comparative binding-pattern analysis with daidzin, rather than as a validated benchmark of docking reproducibility.

### Molecular dynamics simulation

Molecular dynamics simulations were carried out in GROMACS v2024.4 using the CHARMM36 force field under physiological conditions [27]. Protein coordinates were curated in Swiss-PdbViewer to address missing atoms or residues [28], and ligand topologies for daidzin and sirtinol were generated with SwissParam [29]. The complex was placed in a periodic cubic box with a minimum solute to edge distance of 1.0 nm, solvated with Transferable Intermolecular Potential 3-Point (TIP3P) water, and neutralized with Na⁺ and Cl^-^ to an ionic strength of 0.15 M [28–30]. Energy minimization employed the steepest descent integrator for 50,000 steps with emtol = 1000.0 and emstep = 0.01, using the Verlet cutoff scheme with a grid-based neighbor search, nstlist = 1, rlist = 1.2 nm, Particle Mesh Ewald (PME) electrostatics with rcoulomb = 1.2 nm, and van der Waals interactions treated by cutoff with a force-switch modifier where rvdw-switch = 1.0 nm and rvdw = 1.2 nm, while Dispersion Correction (DispCorr) was disabled. An ion-only minimization used rlist = 1.0 nm, cutoff electrostatics, rcoulomb = 1.0 nm, and rvdw = 1.0 nm. Equilibration comprised 100 ps NVT (Number of particles, Volume, Temperature) and 100 ps NPT (Number of particles, Pressure, Temperature) at 300 K with position restraints, Linear Constraint Solver (LINCS) constraints on h-bonds, V-rescale thermostat coupling groups with tau_t = 0.1 ps, followed by pressure control in NPT using Berendsen coupling at 1 bar with tau_p = 2.0 ps and compressibility = 4.5e^-5^ bar^-1^. Production dynamics used the leap-frog integrator with dt = 2 fs for 200 ns under Parrinello-Rahman pressure coupling at 1 bar, with pme_order = 4 and fourierspacing = 0.16 nm, and coordinates and energies recorded every 10 ps [31,32]. Trajectories were evaluated using root-mean-square deviation (RMSD), root-mean-square fluctuation (RMSF), radius of gyration (Rg), number of hydrogen bonds, and solvent-accessible surface area (SASA), with plotting performed in Grace (Grace Development Team) [33].

### ADMET prediction

ADMET prediction, defined by assessments of Absorption, Distribution, Metabolism, Excretion, and Toxicity, is a core element of early drug discovery and is applied in computational, *in silico*, and *in vitro* contexts to appraise pharmacokinetic performance and safety liabilities. Such evaluation facilitates candidate prioritization, reduces attrition through early identification of unfavorable properties, and guides lead optimization and resource allocation before costly late-stage development and clinical assessment. In the present work, pharmacokinetic and toxicity-related parameters of daidzin and sirtinol were estimated using the pkCSM platform, which bases its predictive models on graph-derived molecular descriptors [34].

### MMGBSA analysis

MMGBSA is an endpoint free-energy approach commonly applied in drug discovery to approximate ligand-protein binding affinities, offering improved reliability relative to docking scoring functions while requiring substantially fewer computational resources than rigorous alchemical frameworks such as free energy perturbation. Binding free energy is obtained from ensemble-averaged energy terms derived from molecular mechanics in vacuum together with polar solvation estimated by the Generalized Born model and non-polar solvation approximated by the surface-area component, calculated across multiple MD snapshots [35]. In this study, MMGBSA calculations were conducted using the gmx_MMPBSA package with the CHARMM36 force field to quantify the binding affinities of daidzin and sirtinol toward the protein associated with the strongest docking interactions. The electrostatic solvation contribution was evaluated through the GB formalism. In contrast, the non-polar term was derived from SASA under the assumption of proportionality to solute-solvent surface tension. For energy decomposition, 125 representative frames were extracted from the final 80 ns segment of the 200 ns trajectory, corresponding to 120–200 ns. This interval was selected because the RMSD profiles indicated that both ligand-protein complexes had reached a stable dynamic regime after the initial equilibration period, allowing MMGBSA calculation from a representative equilibrated trajectory segment. The resulting mean binding free energies reflected the dynamic characteristics of ligand-protein association in MD-derived conformational ensembles and supported comparative assessment of complex stability and affinity.

### Quantum chemistry computation using DFT method

DFT provides an electronic-structure framework that supports drug discovery by characterizing electron distributions, frontier orbital features, and reactivity trends relevant to ligand recognition and potential metabolic liability assessment. Relative to more computationally demanding quantum approaches, DFT can yield quantitative descriptors of electron density and frontier molecular orbitals that assist lead optimization and mechanistic interpretation of ligand-receptor interactions [36]. In this study, the geometries of daidzin and sirtinol were fully optimized using ORCA 6.1.0. Initial structures were generated in Avogadro, and subsequent visualization and orbital inspection were performed in IboView v20211019 [35–38]. All calculations were executed at the B3LYP/6-31G(d,p) level to obtain molecular electronic wavefunctions. Based on the optimized structures, quantum-chemical descriptors were derived, including the highest occupied molecular orbital (HOMO) energy, lowest unoccupied molecular orbital (LUMO) energy, the energy gap (ΔE), chemical potential (µ), electronegativity (χ), hardness (η), softness (σ), and electrophilicity index (ω) [39,40]. These quantities were estimated using Koopmans’ theorem to describe the electronic characteristics and reactivity profiles of the investigated compounds [41,42].

## Results and discussion

### Identification of targets of daidzin and breast cancer

**Fig 2:**
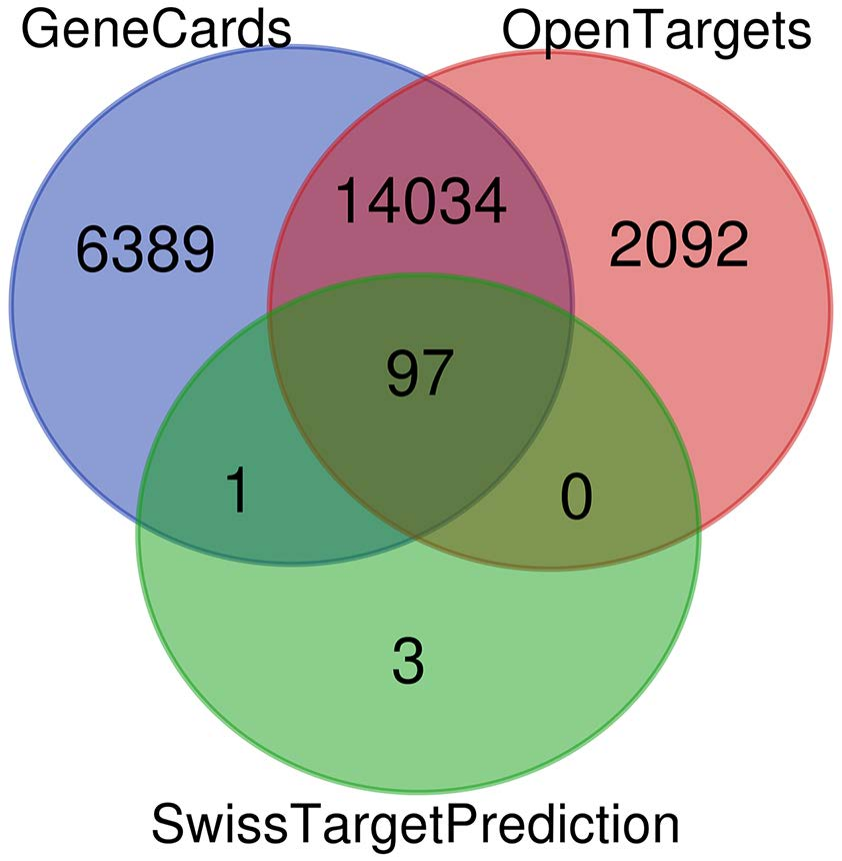
Venn diagram illustrating the common targets between daidzin and breast cancer.

Network pharmacology, particularly in research on traditional medicine, provides a systems-level, data-driven approach to characterize the complex pharmacological behavior of herbal prescriptions, in which multiple constituents may act on numerous molecular targets concurrently. This framework supports a transition from reductionist, single-target assumptions to network-oriented target models that more accurately reflect biological interconnectivity. By integrating bioinformatics, systems biology, and artificial intelligence methods, network pharmacology facilitates the identification of putative bioactive constituents, the prediction and prioritization of therapeutic targets, and the mechanistic assessment of candidate interventions for multifactorial diseases [43]. Using SwissTargetPrediction, 101 putative molecular targets of daidzin were obtained. Breast cancer-associated targets were collected from GeneCards (20,521 targets) and Open Targets (16,223 targets). Following deduplication and integration with breast cancer-related targets, 97 common targets were retained, indicating a substantive overlap between predicted daidzin targets and genes implicated in breast cancer (Fig 2).

To further characterize functional relationships among these candidates, a protein-protein interaction network was constructed to delineate inter-protein connectivity and to support interpretation of their potential involvement in cellular functions and disease-relevant pathways.

### Construction of protein-protein interaction

**Fig 3:**
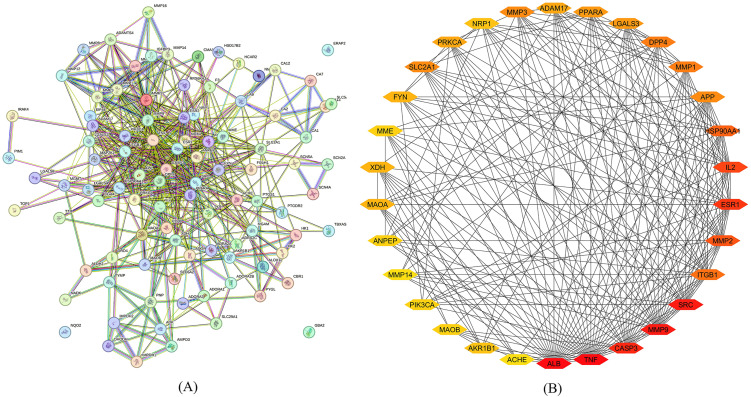
The protein-protein interaction network of anti-breast cancer targets associated with daidzin (A) and top 30 proteins with the highest degree (B).

Protein-protein interaction analysis was performed to characterize the breast cancer-relevant molecular context of daidzin-associated targets using the STRING database, with network visualization conducted in Cytoscape (Fig 3A). The 97 common targets identified from the daidzin-breast cancer overlap were submitted to STRING as the initial input set. To improve visualization and focus on highly connected candidates, the PPI network presented in Fig 3B was restricted to the top 30 proteins ranked by degree. This 30-node subset therefore represents the most connected portion of the 97 common targets rather than a separate target-identification result.

Hub gene identification was conducted with the CytoHubba plugin using the protein-protein interaction network of the 97 common targets, and 12 topological algorithms were applied to support hub inference. Hub prioritization was based on the degree metric, and the resulting rankings are reported in Table 1, indicating the potential relevance of these candidates to daidzin-associated therapeutic interactions in breast cancer.

**Table 1:** Core targets associated with daidzin for breast cancer therapy.

N°	Name	Degree	Average Shortest Path Length	Closeness Centrality	Clustering Coefficient
1	ALB	28	1.0345	0.9667	0.4841
2	TNF	28	1.0345	0.9667	0.4841
3	MMP9	23	1.2069	0.8286	0.5889
4	CASP3	23	1.2069	0.8286	0.5613
5	SRC	20	1.3103	0.7632	0.6579
6	ITGB1	20	1.3103	0.7632	0.6053
7	MMP2	19	1.3448	0.7436	0.6901
8	ESR1	19	1.3448	0.7436	0.6082
9	IL2	18	1.3793	0.7250	0.6863
10	HSP90AA1	16	1.4483	0.6905	0.6917

The top ten hub genes were ALB, TNF, MMP9, CASP3, SRC, ITGB1, MMP2, ESR1, IL2, and HSP90AA1, each characterized by high degree values. Their prominent network connectivity supports their selection as key candidates that may contribute to the therapeutic interaction landscape of daidzin in breast cancer.

### GO and KEGG pathway enrichment analysis

**Fig 4:**
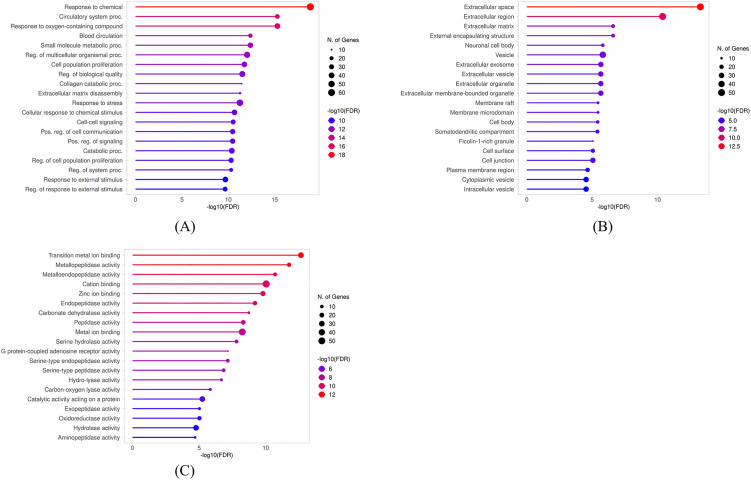
Gene ontology of target genes, including biological process (A), cellular components (B), and molecular function (C).

Gene Ontology enrichment analysis was performed for the 97 common targets to characterize functional annotations across biological process, cellular component, and molecular function. Gene Ontology provides a curated and hierarchically structured vocabulary that supports standardized functional interpretation of gene sets across species [44]. In the biological process category (Fig 4A), 1000 enriched terms were retrieved from the input gene set. The strongest signal corresponded to the chemical response, with 63 annotated genes. Circulatory system process (26 genes), response to oxygen-containing compound (39 genes), and blood circulation (22 genes) were also highly significant, indicating functional convergence on stimulus responsiveness and vascular or perfusion-related biology that is frequently implicated in breast tumor progression and therapy response through angiogenic and microenvironmental mechanisms [45]. Enrichment was also observed for small-molecule metabolic process (35 genes) and multiple communication and signaling terms, including positive regulation of cell communication (34 genes) and positive regulation of signaling (34 genes), consistent with the centrality of intercellular signaling circuits within the tumor microenvironment [46]. Notably, extracellular matrix remodeling was represented by collagen catabolic process (10 genes) and extracellular matrix disassembly (11 genes), supporting the relevance of matrix turnover to invasive phenotypes in breast cancer [47].

For cellular component enrichment, the most significant localizations were extracellular space (50 genes) and extracellular region (52 genes), followed by extracellular matrix (16 genes) and external encapsulating structure (16 genes) (Fig 4B). Vesicle-associated categories were also prominent, including vesicle (43 genes), extracellular exosome (29 genes), extracellular vesicle (29 genes), and cytoplasmic or intracellular vesicle (29 genes). This distribution supports an interpretation in which secreted and vesicle-mediated compartments contribute substantially to the functional landscape of the gene set, aligned with evidence that extracellular vesicles participate in breast cancer cell communication and microenvironmental modulation [48]. Enrichment for membrane raft (11 genes) and membrane microdomain (11 genes) further suggests the involvement of specialized membrane organization that can coordinate signaling receptor dynamics in cancer [49].

As shown in Fig 4C, metal-associated functions dominated the most significant terms, including transition metal ion binding (31 genes), cation binding (53 genes), zinc ion binding (20 genes), and metal ion binding (40 genes). Proteolysis-related activities were strongly represented by metallopeptidase activity (15 genes), metalloendopeptidase activity (12 genes), and additional endopeptidase and peptidase functions, which align with the biological process enrichment for extracellular matrix disassembly and the documented contribution of protease networks, including MMP2 and MMP9, to breast cancer invasion and prognosis [11]. Carbonate dehydratase activity exhibited marked enrichment (6 genes), consistent with the established role of carbonic anhydrase biology in regulating tumor pH adaptation and progression, and with ongoing therapeutic interest in CAIX-directed strategies [50]. In addition, G protein-coupled adenosine receptor activity was enriched (4 genes), consistent with reports that adenosine signaling can reflect immunosuppressive microenvironmental states in breast cancer [51]. For visualization, the top 20 terms in each GO domain were selected from the enrichment outputs and displayed as lollipop plots, with statistical significance represented as minus log10 of the FDR and marker size proportional to the number of mapped genes, using FDR < 0.05 as the selection threshold.

**Fig 5:**
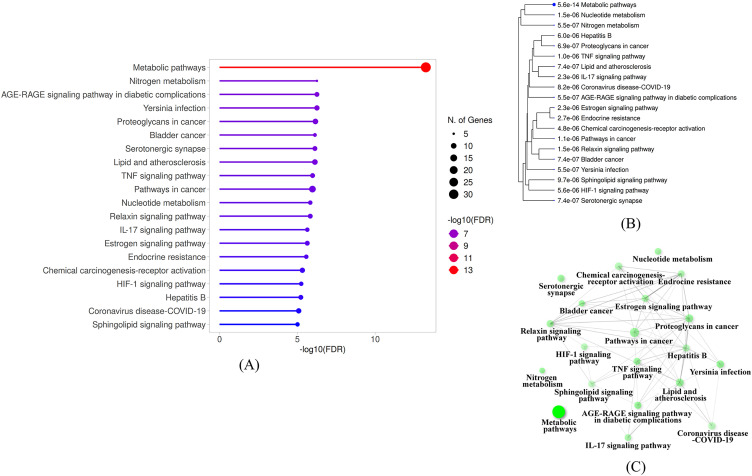
Functional enrichment analysis of targeted genes showing KEGG pathways (A), clustering between pathways (B), and pathways interaction network (C).

KEGG pathway enrichment analysis was performed for the targeted gene set to contextualize the implicated biological programs at the pathway level. The KEGG knowledgebase provides manually curated pathway maps that support functional interpretation of molecular datasets and the reconstruction of pathway-level associations [52]. Of the enrichment outputs, 214 KEGG pathways satisfied the statistical filtering criteria in the provided results. The top 20 terms were visualized as a lollipop plot (Fig 5A), with significance indicated by the negative log10 of the adjusted metric and point size reflecting the number of mapped genes. Among these, metabolic pathways contained the largest mapped gene set with 34 genes, suggesting that the targets converge on metabolic programs that are frequently rewired during breast tumor development and therapeutic adaptation. Additional metabolism-related annotations, including nucleotide metabolism (7 genes) and nitrogen metabolism (5 genes), further supported an enrichment profile consistent with biochemical plasticity in malignant systems. Several cancer-relevant pathway terms were prominent. Pathways in cancer included 14 genes, and proteoglycans in cancer included 10 genes, indicating enrichment of canonical oncogenic circuitry together with extracellular matrix-associated signaling. Proteoglycans are established regulators of tumor-stroma interactions and metastatic behavior through effects on growth factor availability, adhesion, and matrix organization, which is compatible with the extracellular matrix signal also observed in the Gene Ontology analysis [53]. In addition, inflammatory signaling was represented by the TNF signaling pathway (8 genes) and the IL-17 signaling pathway (7 genes). These pathways are mechanistically linked to NF-κB-centered transcriptional programs that shape tumor cell survival, invasion, and microenvironmental inflammation [54,55]. Endocrine and hypoxia-associated signaling also emerged. The estrogen signaling pathway (8 genes) and endocrine resistance (7 genes) were enriched, consistent with the central role of estrogen receptor biology and resistance mechanisms in a substantial fraction of breast cancers [56]. The HIF-1 signaling pathway, involving 7 genes, was also significant, aligning with evidence that hypoxia-inducible signaling contributes to metastatic competence and microenvironmental conditioning in breast tumors [57]. Notably, serotonergic synapse with 8 genes was enriched, supporting a potential interface between neurotransmitter-related signaling and tumor phenotypes, including stemness-associated biology described for serotonergic components in breast cancer [58]. Pathways related to lipid signaling were represented by lipid and atherosclerosis (10 genes) and the sphingolipid signaling pathway (7 genes), consistent with reports that sphingolipid metabolism and signaling influence breast cancer progression and treatment responsiveness [59].

To evaluate higher-order structure among enriched pathways, hierarchical clustering was visualized as a dendrogram (Fig 5B). In this representation, pathways with larger gene-set intersections are positioned more closely, indicating common molecular substrates among cancer, inflammatory, endocrine, and metabolic programs. The pathway interaction network (Fig 5C) further summarized these relationships by connecting pathway nodes when gene overlap met the visualization criterion of at least 20 percent. Within this network, node size reflected the number of annotated genes and node color represented relative enrichment strength, enabling identification of densely connected modules. The resulting topology placed broad oncogenic pathways, such as cancer pathways, in proximity to endocrine-associated terms and inflammatory signaling, consistent with known cross-regulation among estrogen receptor signaling, cytokine cascades, and NF-κB-related transcriptional control in breast cancer pathobiology.

### Molecular docking analysis

The molecular docking analysis evaluated daidzin against ten hub proteins implicated in breast cancer, including ALB (6YG9), TNF (1GVS), MMP9 (6ESM), CASP3 (3PD1), SRC (4F5B), ITGB1 (3T9K), MMP2 (1QIB), ESR1 (4XI3), IL2 (4NEJ), and HSP90AA1 (2QG2). Predicted binding energies ranged from −6.00 to −11.49 kcal/mol, indicating heterogeneous affinity across targets. The most favorable score was observed for MMP9 (6ESM) at −11.49 kcal/mol, followed by TNF (1GVS) at −10.28 kcal/mol and ESR1 (4XI3) at −9.63 kcal/mol. Intermediate affinities were obtained for ALB (6YG9) at −8.24 kcal/mol, MMP2 (1QIB) at −7.78 kcal/mol, CASP3 (3PD1) at −7.46 kcal/mol, and ITGB1 (3T9K) at −7.25 kcal/mol, whereas SRC (4F5B), IL2 (4NEJ), and HSP90AA1 (2QG2) displayed comparatively weaker docking scores (Table 2 and S2 Table).

**Table 2:** The interactions between selected ligands and target genes.

Compound	Target genes	Target PDB ID	Binding energy (kcal/mol)	Hydrogen bonds	Van der Waals interaction	Hydrophobic interaction
Daidzin	MMP9	6ESM	−11.49	Ala189, His226, Pro246, Tyr248, Arg249, Pro255	Gly186, Leu187, Leu188, Gln227, Ala242, Tyr245, Thr251	Leu222, Val223, His226, Leu243, Met247, Tyr248
	TNF	1GVS	−10.28	Pro24, Thr26, Gln100, His184, Tyr186, Arg233, Gln241, Tyr303, Gly323, Arg324	Ala23, Leu25, Ala58, Trp102, His181, Ala300, Gly301, Ala302, Ala321, Phe322, Asp325	Leu275
	ESR1	4XI3	−9.63	Leu346, Arg349, Glu353, Leu387, Lys531, Asn532	Met343, Leu349, Asp351, Trp383, Leu384, Met388, Leu391, Phe404	Thr347, Ala350, Leu525, Cys530, Val533
B9Z	MMP9	6ESM	−10.54	Ala189, Ala191, His226, Gln227, His236, Ala242, Arg249	Gly186, His190, Leu222, His230, Tyr245, Met247	Leu187, Leu188, Val223, His226, Leu243, Tyr248, Arg249
Sirtinol	MMP9	6ESM	−10.59	Pro246	Gly186, Leu187, Ala189, His190, Tyr218, Leu222, Gln227, His230, His236, Met247, Arg249	Leu188, Val223, His226, Tyr248

**Fig 6:**
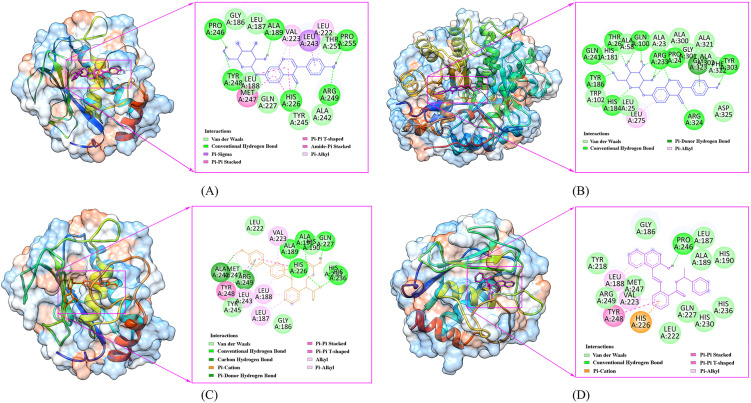
Modelling the interaction diagram of docking complexes. (A) Daidzin-6ESM, (B) Daidzin-1GVS, (C) B9Z-6ESM, and (D) Sirtinol-6ESM.

As shown in Fig 6, the daidzin-MMP9 complex exhibited a dense interaction pattern within the binding pocket. Hydrogen bonding involved Ala189, His226, Pro246, Tyr248, Arg249, and Pro255, complemented by van der Waals contacts with Gly186, Leu187, Leu188, Gln227, Ala242, Tyr245, and Thr251, and hydrophobic contacts with Leu222, Val223, His226, Leu243, Met247, and Tyr248. The recurrent participation of His226 and the surrounding hydrophobic residues suggests occupancy of a catalytically relevant cleft in the MMP9 domain, a feature consistent with the established role of gelatinases in extracellular matrix remodeling and metastatic dissemination [60]. In breast cancer, elevated MMP2 and MMP9 expression has been associated with adverse clinical outcomes, which supports prioritization of gelatinase-centered mechanisms in computational target validation. Daidzin also demonstrated strong docking scores against TNF and ESR1, two nodes frequently linked to inflammatory signaling and endocrine response biology in breast malignancy. The TNF complex (−10.28 kcal/mol) involved multiple polar contacts within a broad residue set, consistent with the pleiotropic influence of TNF family signaling on tumor progression and the tumor microenvironment. The ESR1 complex (−9.63 kcal/mol) mapped to the canonical ligand-binding region, aligning with the central role of estrogen receptor signaling and endocrine resistance in hormone receptor-positive breast cancer.

Beyond these three targets, daidzin also demonstrated quantifiable binding to ITGB1, SRC, CASP3, IL2, and HSP90AA1, with predicted binding energies of −7.25 kcal/mol for ITGB1 (3T9K), −6.15 kcal/mol for SRC (4F5B), −7.46 kcal/mol for CASP3 (3PD1), −6.54 kcal/mol for IL2 (4NEJ), and −6.00 kcal/mol for HSP90AA1 (2QG2). The interaction patterns indicate that these complexes are supported by multiple contact categories rather than by a single dominant force. For ITGB1, the docking pose involved 6 residues in hydrogen bonding interactions, 8 in van der Waals contacts, and 3 in hydrophobic contacts, consistent with a mixed polar and nonpolar interface. For SRC, the interaction footprint was smaller, comprising 3 hydrogen-bond residues, 6 van der Waals residues, and 1 hydrophobic residue, consistent with its lower docking score relative to the other candidates. CASP3 exhibited 7 hydrogen-bond residues, 5 van der Waals residues, and 3 hydrophobic residues, reflecting a comparatively balanced interaction profile that may stabilize ligand association within a functionally relevant region. IL2 showed interactions with 8 hydrogen-bonding residues, 2 van der Waals residues, and 4 hydrophobic residues, suggesting a greater relative contribution of polar contacts. HSP90AA1 showed 6 hydrogen-bonding residues, an extensive set of 9 van der Waals interactions, and 3 hydrophobic residues, indicating broad surface complementarity despite a less favorable binding energy. Collectively, these quantitative interaction fingerprints support the interpretation that, in addition to the higher-affinity targets, daidzin retains the capacity to engage proteins involved in cell adhesion and invasion biology through ITGB1, kinase-linked oncogenic signaling through SRC, apoptosis regulation through CASP3, immune modulation through IL2, and chaperone-dependent proteostasis through HSP90AA1, thereby providing mechanistic breadth across multiple hallmarks relevant to breast cancer (S1 Fig) [59–63].

For MMP9, B9Z was included because it is the co-crystallized inhibitor in the selected 6ESM structure and therefore provides a more appropriate reference ligand for this target. B9Z showed a binding energy of −10.54 kcal/mol and formed hydrogen bonds with Ala189, Ala191, His226, Gln227, His236, Ala242, and Arg249. Sirtinol was retained only as a general breast cancer-related comparator and not as an MMP9-specific positive control. The docking score against 6ESM was −10.59 kcal/mol, with a hydrogen bond involving Pro246. Under the same docking conditions, daidzin showed the most favorable score among the three ligands, with a binding energy of −11.49 kcal/mol. This value was 0.95 kcal/mol lower than that of B9Z and 0.90 kcal/mol lower than that of sirtinol. Daidzin also shared key pocket residues with B9Z, including Ala189, His226, and Arg249, while forming additional hydrogen-bond contacts with Pro246, Tyr248, and Pro255. The stronger docking score and broader interaction profile support preferential accommodation of daidzin within the MMP9 binding pocket and justify selection of the daidzin-6ESM complex for subsequent molecular dynamics simulation.

Accordingly, the MMP9-centered docking results were interpreted primarily through comparison with B9Z as the target-relevant reference ligand, whereas sirtinol was used only to maintain continuity with previous breast cancer-related docking analyses. Comparison with the previous genistin docking analysis indicates a shift in the top-ranked target. In the genistin analysis, SIRT1 (4KXQ) was reported as the strongest complex, with a binding energy of −10.72 kcal/mol, while sirtinol (4KXQ) served as a comparator with a binding energy of −10.56 kcal/mol. In contrast, the present daidzin dataset identifies MMP9 (6ESM) as the most favorable target, with a binding energy of −11.49 kcal/mol. For common biological targets, daidzin yielded more favorable docking scores than genistin for TNF (−10.28 kcal/mol vs. −9.95 kcal/mol) and for ESR1 (−9.63 kcal/mol vs. −9.17 kcal/mol). These differences suggest that daidzin may preferentially engage interfaces associated with inflammatory signaling and estrogen receptor biology. In contrast, genistin showed its strongest predicted affinity for a regulator linked to cellular metabolism and stress responses [64–66]. This divergence is compatible with the concept that structurally related isoflavone glycosides can generate target-specific interaction fingerprints that redistribute pathway emphasis across extracellular matrix remodeling, cytokine networks, and endocrine regulation.

Compared with prior experimental evidence on the aglycone daidzein, which has been reported to induce apoptosis in MCF-7 cells through mitochondrial-associated and caspase-dependent mechanisms, the present docking results suggest that daidzin may exert similar antitumor effects after intestinal hydrolysis to daidzein *in vivo*. In this dataset, daidzin exhibited its strongest predicted affinity toward MMP9 (6ESM) with a binding energy of −11.49 kcal/mol, accompanied by a dense interaction footprint within the binding pocket, which is biologically consistent with the adverse prognostic associations of MMP9 overexpression in breast cancer and its role in extracellular matrix remodeling. Notably, a comparable computational approach has previously advanced daidzein-MMP9 complexes to molecular dynamics simulations, supporting the methodological rationale for dynamic validation at this target [67]. Given these considerations, daidzin-6ESM was selected for molecular dynamics simulations, with sirtinol-6ESM retained as a comparative control to assess complex stability and interaction persistence.

### Molecular dynamics simulations

Molecular dynamics simulation is a computational framework that describes the time-dependent behavior of atomic systems by numerically integrating Newton’s equations of motion. Within this formalism, atoms are represented as interacting particles governed by an empirical force field, enabling quantitative examination of conformational fluctuations, ligand-receptor association, and structural adaptation in chemical and biomolecular assemblies at femtosecond-scale time resolution [68]. Accordingly, molecular dynamics simulations were conducted for the daidzin complex, with sirtinol included as a reference ligand, and the trajectories were evaluated using RMSD, RMSF, Rg, Hbonds, and SASA. For the daidzin-6ESM, the total energy and potential energy were −193,613 and −243,256 kJ/mol, respectively. For the sirtinol-6ESM, the corresponding total energy and potential energy were −199,112 and −243,746 kJ/mol, respectively.

The RMSD trajectories of both complexes show a rapid adaptation phase at the beginning of the simulation. RMSD increases from approximately 0.15 to 0.20 nm to the vicinity of 0.40 nm within the first 10–15 ns, which is consistent with relaxation from the docking pose and local rearrangement of the binding environment in solution. After this equilibration period, the RMSD for both systems remains predominantly within the 0.40–0.50 nm range for most of the trajectory, indicating that the overall protein framework reaches a dynamically stable regime with moderate fluctuations. A minor divergence appears at late time points. The sirtinol-6ESM complex shows a tendency toward lower RMSD values after approximately 150 ns, decreasing to approximately 0.38–0.43 nm. In contrast, the daidzin-6ESM complex remains around 0.43–0.48 nm, without a clear downward trend. This pattern supports the view that both systems are globally stable, whereas the late differences likely reflect distinct microstructural equilibria within the bound state [69].

**Fig 7:**
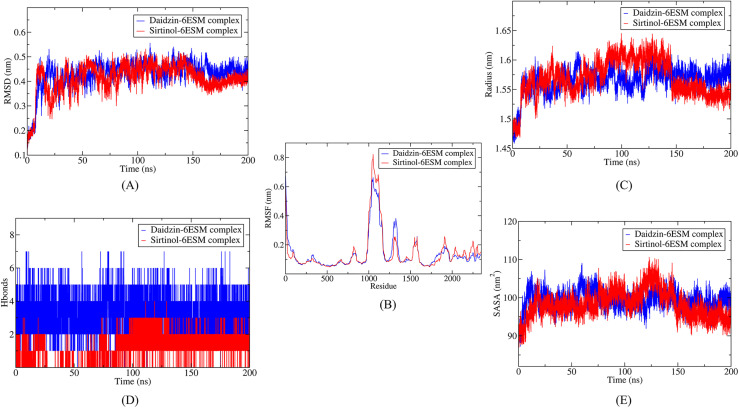
Results of MD simulation for the bindings of daidzin (blue) and sirtinol (red) with the protein 6ESM. (A) RMSD, (B) RMSF, (C) Rg, (D) Hbonds, and (E) SASA.

Within the catalytically relevant segment spanning Gly112 to Gly269, the RMSF profiles indicate that both complexes exhibit low-amplitude positional fluctuations across most residues, consistent with a structurally restrained binding region during the 200 ns trajectory. In this interval, the overall RMSF patterns of the daidzin-6ESM and sirtinol-6ESM systems are broadly comparable, suggesting that ligand association does not induce pronounced destabilization of the local backbone framework (Fig 7B). The residues that form the canonical pocket environment, including the clustered histidines and adjacent hydrophobic and polar positions in the His190-His236 region, exhibit limited flexibility relative to the highly mobile peripheral segments of the protein, supporting a stable microenvironment around the ligand-binding cavity. Minor local increases in fluctuation are confined to short loop-like stretches within the same interval and do not propagate into a sustained elevation across the entire Gly112-Gly269 region. Notably, the sirtinol-6ESM trace shows slightly higher local mobility at selected positions within this range when compared with daidzin-6ESM, whereas daidzin-6ESM generally preserves a more restrained fluctuation profile in the binding-region residues, consistent with the denser polar interaction pattern observed for daidzin in the simulation.

The Rg trajectories for both systems rise rapidly from approximately 1.47 to 1.50 nm to about 1.55 nm during the early segment of the simulation, consistent with structural relaxation and redistribution in a solvated thermally fluctuating state. During the mid portion of the trajectory, daidzin-6ESM fluctuates mainly around 1.55 to 1.60 nm. The sirtinol-6ESM complex exhibits a more pronounced expansion phase between roughly 80 and 140 ns, often reaching 1.60–1.64 nm. After approximately 150 ns, sirtinol-6ESM transitions to a more compact regime, with Rg decreasing to approximately 1.52 to 1.56 nm (Fig 7C). This pattern is compatible with a late conformational transition in the sirtinol system toward a more compact state. In contrast, daidzin maintains a comparatively persistent compactness profile without a clear late compaction phase.

The hydrogen bond counts show a consistent difference between ligands throughout the 200 ns simulation. The daidzin-6ESM complex maintains a higher number of hydrogen bonds, typically 3−5, with occasional transient increases to 6−7. In contrast, sirtinol-6ESM remains primarily within 0–2, with a short interval around 100–130 ns reaching approximately 2–3 (Fig 7D). Hydrogen bonds often contribute to the directional anchoring of a ligand within a binding pocket. They can support persistent association in aqueous environments, although overall stability also depends on hydrophobic and electrostatic contributions [70]. These results support a stronger and more sustained polar interaction component for daidzin relative to sirtinol within the 6ESM binding site, which is consistent with the lower amplitude of the central RMSF peak for daidzin compared with sirtinol.

SASA increases rapidly for both systems from approximately 88–92 nm² to about 95–105 nm² in the early phase, reflecting adjustments in solvent-exposed surface area as the system reaches initial dynamic equilibrium. The daidzin-6ESM complex fluctuates between approximately 98 and 103 nm², with relatively stable behavior. The sirtinol-6ESM complex shows a marked transient increase in SASA around 120–135 ns, reaching close to 110 nm², followed by a decrease to approximately 92–98 nm² after 150 ns (Fig 7E). This SASA trend aligns with the Rg behavior, in which sirtinol shows a mid-trajectory expansion followed by late compaction. The weaker phase-like variation for daidzin suggests that the solvent-exposed surface of the complex is less affected by large-scale structural rearrangements, supporting a relatively stable ligand-protein interfacial organization across the trajectory [71].

The MMP9 structure used for molecular dynamics simulations was retrieved from the Protein Data Bank under accession code 6ESM, which captures an inhibitor-bound conformation and therefore provides a well-defined binding cavity for stability assessment. From a disease perspective, MMP9 has been associated with unfavorable breast cancer prognosis and participates in extracellular matrix remodeling, which can support invasive and metastatic phenotypes. Across the 200 ns trajectories, both complexes exhibited global structural stability, as indicated by RMSD behavior. Nevertheless, daidzin-6ESM maintained higher hydrogen bond counts and exhibited a SASA profile with reduced phase-like shifts compared with sirtinol-6ESM, suggesting a more persistent interfacial organization during the simulation. Collectively, these results support prioritization of the daidzin-6ESM complex for downstream post-simulation analyses or extended simulations, while sirtinol-6ESM remains appropriate as a comparative control for stability benchmarking under solvated conditions.

### MMGBSA analysis

Binding free energies for the daidzin-6ESM and sirtinol-6ESM complexes were estimated using an end-state MMGBSA framework, which approximates the binding free energy as the difference between the free energy of the complex and the sum of the free energies of the separated protein and ligand, with decomposition into gas-phase and solvation contributions [35]. In this approach, the gas-phase term is commonly expressed as ΔG_GAS = ΔVDWAALS + ΔEEL, whereas the solvation term is represented as ΔG_SOLV = ΔEGB + ΔESURF, and the total estimate is ΔTOTAL = ΔG_GAS + ΔG_SOLV [72]. As detailed in Table 3, daidzin-6ESM exhibited a substantially more favorable mean binding free energy than sirtinol-6ESM, with ΔTOTAL of −46.86 ± 3.83 kcal/mol compared with −14.12 ± 8.99 kcal/mol, indicating stronger predicted association and lower dispersion across the sampled ensemble for daidzin.

**Table 3:** Free energy of binding obtained using MMGBSA calculations.

Energy Component	Average (kcal/mol)		Standard Deviation	
	Daidzin-6ESM	Sirtinol-6ESM	Daidzin-6ESM	Sirtinol-6ESM
ΔVDWAALS	−54.30	−21.55	2.54	11.72
ΔEEL	−31.36	−5.75	7.23	7.96
ΔEGB	45.22	15.91	5.55	10.40
ΔESURF	−6.43	−2.74	0.33	1.44
ΔGGAS	−85.65	−27.30	6.69	16.89
ΔGSOLV	38.79	13.18	5.54	9.29
ΔTOTAL	−46.86	−14.12	3.83	8.99

The energetic preference for daidzin was driven by markedly stronger nonbonded interactions in the gas phase. Specifically, ΔVDWAALS was −54.30 ± 2.54 kcal/mol for daidzin versus −21.55 ± 11.72 kcal/mol for sirtinol, identifying van der Waals packing as the dominant favorable contributor [73]. In parallel, electrostatic interactions further reinforced binding for daidzin, with ΔEEL of −31.36 ± 7.23 kcal/mol compared with −5.75 ± 7.96 kcal/mol for sirtinol. This combination yielded a more favorable ΔG_GAS for daidzin at −85.65 ± 6.69 kcal/mol relative to −27.30 ± 16.89 kcal/mol for sirtinol.

Both complexes showed opposing desolvation penalties, consistent with the well-recognized compensation between favorable Coulombic terms and unfavorable polar solvation in MMGBSA analyses [74]. For daidzin-6ESM, the polar solvation component ΔEGB was 45.22 ± 5.55 kcal/mol, and the nonpolar surface term ΔESURF was −6.43 ± 0.33 kcal/mol, giving ΔG_SOLV of 38.79 ± 5.54 kcal/mol. For sirtinol-6ESM, ΔEGB and ΔESURF were 15.91 ± 10.40 kcal/mol and −2.74 ± 1.44 kcal/mol, respectively, yielding ΔG_SOLV of 13.18 ± 9.29 kcal/mol. Although daidzin incurred a larger solvation penalty, this cost was more than offset by its stronger van der Waals and electrostatic contributions, resulting in the more favorable ΔTOTAL [75].

Overall, the MMGBSA decomposition supports the conclusion that daidzin forms a more stable energetic association with the 6ESM target than sirtinol, primarily through enhanced dispersion and electrostatic interactions, with the lower standard deviation of ΔTOTAL also indicating greater energetic consistency across the analyzed conformational ensemble. These characteristics are consistent with the typical utility of end-state free energy methods for comparative ranking of ligand binding strength, provided appropriate awareness of entropy omission and model dependence.

### ADMET parameters

ADMET profiling for daidzin and sirtinol was generated using the pkCSM prediction framework, which applies graph-based signatures to estimate key pharmacokinetic and toxicity endpoints relevant to early compound prioritization. Table 4 summarizes the pkCSM-predicted ADMET parameters for daidzin and sirtinol, providing a comparative overview of their absorption, distribution, metabolism, excretion, and toxicity profiles.

**Table 4:** Predicted ADMET properties of daidzin and sirtinol.

ADMET properties	Unit	Daidzin	Sirtinol
Water Solubility	(Log mol/L)	−2.784	−4.120
Cancer Colon-2 (Caco2) permeability	(Log Papp in 10^−6^ cm/s)	0.240	0.934
Intestinal absorption (Human)	(% Absorbed)	59.319	92.147
Skin permeability	(Log Kp)	−2.736	−2.735
P-glycoprotein substrate	Yes/No	Yes	Yes
P-glycoprotein I inhibitor	Yes/No	No	Yes
P-glycoprotein II inhibitor	Yes/No	No	Yes
Volume of Distribution at Steady State (VDss)	(Log L/kg)	−0.166	−0.706
Fraction unbound (human)	(Fu)	0.199	0
Blood-brain barrier (BBB) permeability	(Log BB)	−1.232	−0.122
Central nervous system (CNS) permeability	(Log PS)	−3.584	−1.494
Cytochrome P450 Family 2 Subfamily D Member 6 (CYP2D6) substrate	Yes/No	No	No
Cytochrome P450 Family 3 Subfamily A Member 4 Substrate (CYP3A4) substrate	Yes/No	No	Yes
Cytochrome P450 Family 1 Subfamily A Member 2 Inhibitor (CYP1A2) inhibitor	Yes/No	No	Yes
Cytochrome P450 Family 2 Subfamily C Member 19 Inhibitor (CYP2C19) inhibitor	Yes/No	No	Yes
Cytochrome P450 Family 2 Subfamily C Member 9 Inhibitor (CYP2C9) inhibitor	Yes/No	No	Yes
Cytochrome P450 Family 2 Subfamily D Member 6 Inhibitor (CYP2D6) inhibitor	Yes/No	No	No
Cytochrome P450 Family 3 Subfamily A Member 4 Inhibitor (CYP3A4) inhibitor	Yes/No	No	Yes
Total clearance	(Log ml/min/kg)	0.104	0.097
Renal Organic Cation Transporter 2 (OCT2) substrate	Yes/No	No	No
AMES toxicity	Yes/No	No	Yes
Max. tolerated dose (human)	(Log mg/kg/day)	0.488	0.119
Human Ether-à-go-go-Related Gene (hERG) I inhibitor	Yes/No	No	No
hERG II inhibitor	Yes/No	Yes	Yes
Oral rat acute toxicity (LD50)	(mol/kg)	2.738	2.363
Oral rat chronic toxicity (LOAEL)	(Log mg/kg_bw/day)	4.717	0.669
Hepatotoxicity	Yes/No	No	Yes
Skin sensitization	Yes/No	No	No
*Tetrahymena pyriformis* toxicity	(Log ug/L)	0.285	0.295
Minnow toxicity	(Log mM)	3.902	−0.192

Daidzin demonstrated higher predicted aqueous solubility than sirtinol, with water solubility values of −2.784 and −4.120 log mol/L, respectively, indicating a less solubility-limited profile for daidzin under the applied model. Predicted intestinal permeability favored sirtinol, with Caco2 permeability values of 0.934 compared with 0.240 log Papp (10^−6^ cm/s) for daidzin, consistent with the higher predicted human intestinal absorption of sirtinol (92.147%) relative to daidzin (59.319%). Skin permeability was similar for both compounds, with log Kp values of −2.736 for daidzin and −2.735 for sirtinol, suggesting comparably restricted dermal penetration. Both compounds were predicted as P glycoprotein substrates, while sirtinol was additionally predicted as a P glycoprotein I inhibitor and P glycoprotein II inhibitor, a pattern compatible with altered efflux-mediated exposure and a higher transporter-mediated interaction liability for sirtinol [76].

The predicted steady-state volume of distribution was higher for daidzin (−0.166 log L/kg) than for sirtinol (−0.706 log L/kg), suggesting a relatively broader peripheral distribution for daidzin within the model’s constraints. The predicted fraction unbound in human plasma was 0.199 for daidzin and 0 for sirtinol, consistent with markedly higher protein binding for sirtinol and a reduced freely available drug fraction. Central distribution estimates differed substantially. Daidzin exhibited low BBB permeability with log BB = −1.232, whereas sirtinol showed a less restricted estimate (log BB = −0.122). For CNS permeability, daidzin presented log PS = −3.584, while sirtinol presented log PS = −1.494. Under commonly used interpretive criteria, log BB < −1 supports poor BBB penetration, and log PS < −3 supports negligible CNS permeability. In contrast, higher log PS values indicate greater CNS access, aligning with limited CNS exposure to daidzin and greater CNS accessibility to sirtinol [77].

Neither compound was predicted as a CYP2D6 substrate. Daidzin was predicted not to be a CYP3A4 substrate, whereas sirtinol was expected to be a CYP3A4 substrate. Inhibition liabilities differed markedly, with daidzin predicted as a non-inhibitor for CYP1A2, CYP2C19, CYP2C9, CYP2D6, and CYP3A4, while sirtinol was expected as an inhibitor of CYP1A2, CYP2C19, CYP2C9, and CYP3A4. This pattern indicates a higher risk of CYP-mediated drug interaction potential for sirtinol, consistent with established pharmacology that CYP inhibition represents a major mechanism underlying clinically relevant drug interactions [78].

Predicted total clearance values were similar, 0.104 for daidzin and 0.097 log ml/min/kg for sirtinol, suggesting comparable overall elimination rates within model uncertainty. Both compounds were predicted to be non-substrates of renal OCT2, indicating that OCT2-mediated renal secretion is unlikely to dominate clearance for either ligand in this dataset.

Toxicity and safety-related endpoints. Daidzin was predicted to be AMES negative, whereas sirtinol was predicted to be AMES positive, indicating a more favorable genotoxicity screening profile for daidzin under the applied *in silico* endpoint. The expected maximum tolerated dose in humans was higher for daidzin (0.488 log mg/kg/day) than for sirtinol (0.119 log mg/kg/day). For cardiotoxicity-related signals, both compounds were predicted as hERG I non-inhibitors and hERG II inhibitors, supporting the need for electrophysiology-oriented confirmation if progression is intended [79]. Acute oral toxicity in rats was higher for daidzin with an LD50 = 2.738 mol/kg relative to sirtinol (2.363 mol/kg), consistent with lower acute toxicity for daidzin when interpreted as a higher tolerated dose. Chronic toxicity predictions also favored daidzin, with LOAEL = 4.717 log mg/kg_bw/day compared with 0.669 for sirtinol. Hepatotoxicity was predicted as negative for daidzin and positive for sirtinol. Skin sensitization was predicted as negative for both compounds. Environmental toxicity-related estimates were similar for *T. pyriformis* (0.285 for daidzin and 0.295 log ug/L for sirtinol). In contrast, minnow toxicity differed strongly, with 3.902 log mM for daidzin and −0.192 log mM for sirtinol, indicating substantially lower predicted aquatic toxicity for daidzin under the pkCSM endpoint definition [80].

The ADMET profile suggests a trade-off between permeability and safety signals. Sirtinol shows stronger predicted intestinal permeability and absorption but also exhibits transporter inhibition, multiple CYP inhibition flags, AMES positivity, and predicted hepatotoxicity. Daidzin shows improved predicted solubility, reduced CYP and transporter inhibition liabilities, AMES negativity, and more favorable chronic toxicity indices, accompanied by lower predicted intestinal absorption and negligible CNS exposure. These contrasts suggest that daidzin has more favorable predicted safety-related flags than sirtinol, but this advantage is accompanied by lower predicted permeability and intestinal absorption. Therefore, the ADMET profile should be interpreted as a safety-exposure trade-off rather than as uniformly favorable developability, and absorption-limited exposure may require formulation or delivery optimization if oral systemic bioavailability is a key objective.

### Quantum chemistry computation using DFT method

Density functional theory is a quantum-mechanical framework routinely employed in modern computer-aided drug design and pharmaceutical sciences due to its favorable balance between accuracy and computational cost. In addition to frontier orbital energies, DFT supports conceptual reactivity analysis through global descriptors derived from EHOMO and ELUMO, including the energy gap ΔE, chemical potential µ, electronegativity χ, global hardness η, softness σ, and electrophilicity index ω, which facilitate interpretation of molecular stability and propensity for charge transfer in ligand-receptor recognition [36]. Table 5 presents the computed frontier orbital energies and conceptual DFT descriptors for daidzin and sirtinol, enabling a quantitative comparison of electronic structure features relevant to intermolecular recognition.

**Table 5:** Quantum descriptors of daidzin and sirtinol.

Molecule	EHOMO (eV)	ELUMO (eV)	ΔE (eV)	µ (eV)	χ (eV)	η (eV)	σ (eV^-1^)	ω (eV)
Daidzin	−8.7123	1.9091	10.6214	−3.4016	3.4016	5.3107	0.1883	1.0894
Sirtinol	−7.8056	1.5487	9.3543	−3.1285	3.1285	4.6772	0.2138	1.0463

EHOMO (eV), highest occupied molecular orbitals; ELUMO (eV), lowest unoccupied molecular orbitals; ΔE (eV), energy gap; µ (eV), chemical potential; χ (eV), electronegativity; η (eV), hardness; σ (eV^-1^), softness; ω (eV), electrophilicity index.

**Fig 8:**
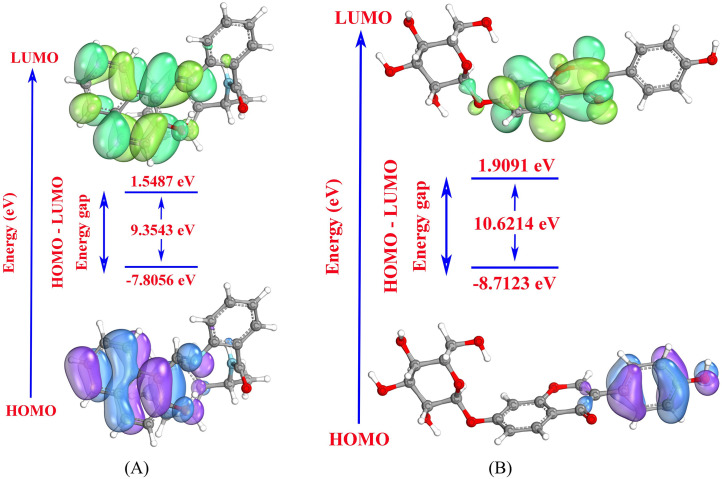
HOMO and LUMO surface diagrams of daidzin (A) and sirtinol (B).

As detailed in Table 5, frontier orbital analysis indicates that daidzin exhibits a more negative EHOMO than sirtinol (−8.7123 versus −7.8056 eV), consistent with reduced electron-donating propensity and increased resistance to oxidation relative to the comparator. In parallel, sirtinol presents a lower ELUMO (1.5487 eV) than daidzin (1.9091 eV), suggesting a comparatively enhanced electron acceptance tendency for sirtinol. The HOMO-LUMO gap further differentiates the compounds, with daidzin displaying a larger ΔE (10.6214 eV) than sirtinol (9.3543 eV). In conceptual DFT, a larger ΔE is generally associated with greater kinetic stability and diminished global reactivity. In contrast, a smaller ΔE indicates higher polarizability and greater facilitation of charge-transfer processes (Fig 8).

The global reactivity descriptors follow the same pattern. Daidzin demonstrates higher hardness η (5.3107 eV) than sirtinol (4.6772 eV), while sirtinol shows higher softness σ (0.2138 eV^-1^) than daidzin (0.1883 eV^-1^) [81]. These relationships support a comparatively more reactive electronic distribution for sirtinol and a more rigid electron density for daidzin. Electronegativity values indicate a stronger electron-attracting character for daidzin (χ 3.4016 eV) relative to sirtinol (χ = 3.1285 eV), with chemical potentials reflecting the corresponding sign convention (µ = −3.4016 versus −3.1285 eV). The electrophilicity index is slightly higher for daidzin (ω 1.0894 eV) than for sirtinol (ω 1.0463 eV), indicating a modestly greater global electrophilic character within the descriptor framework used [82].

Collectively, the descriptor set supports an interpretation in which daidzin is electronically more stable and more rigid. In contrast, sirtinol is softer, with a narrower frontier gap, differences that may influence the balance between stability and charge-transfer contributions during protein binding.

### Limitations of the study

Despite the identification of favorable signals in the computational approach, experimental validation remains essential to confirm the reliability and biological relevance of the predicted daidzin-target interactions. Owing to limitations in equipment availability and related resources, the present work did not include *in vitro* or *in vivo* experimentation. An additional limitation concerns the metabolic fate of daidzin. As a glycosylated isoflavone, daidzin can undergo hydrolysis to daidzein through intestinal or microbial metabolism. Therefore, the predicted interactions reported here reflect daidzin under the applied computational conditions, whereas *in vivo* effects may also involve daidzein or other metabolites formed after biotransformation. Future studies should therefore prioritize biochemical assays to confirm MMP9 inhibitory activity, cell-based assays to evaluate anti-invasive and anti-migratory effects, and breast cancer subtype-specific models to determine the biological relevance of the predicted daidzin-MMP9 interaction. Comparative testing of daidzin and daidzein would also help distinguish whether any observed MMP9-related or anti-invasive effects are attributable to daidzin itself, daidzein, or both compounds. The current lack of experimental evidence, together with the absence of evaluation for unintended covalent inhibition, represents a key limitation that may affect the interpretation of the *in silico* results. An additional methodological limitation concerns the absence of redocking validation for the MMP9 receptor (6ESM). Although B9Z, the co-crystallized ligand, was used as a reference for comparison with daidzin, no heavy-atom RMSD was calculated between the redocked and native crystallographic pose of B9Z to confirm docking grid reliability. Consequently, the reproducibility of the docking protocol for this target was not empirically validated through pose-reproduction analysis, and the MMP9 docking results should be interpreted with this caveat in mind.

## Conclusions

This study provides an integrated computational assessment of daidzin as a candidate modulator of breast cancer-relevant molecular programs, combining network pharmacology with molecular docking, molecular dynamics, MMGBSA, ADMET prediction, and DFT descriptors. Network analysis identified 97 common targets, and hub prioritization highlighted ALB, TNF, MMP9, CASP3, SRC, ITGB1, MMP2, ESR1, IL2, and HSP90AA1. At the same time, GO and KEGG enrichment supported convergence on extracellular and vesicle-associated compartments, metal-ion binding, and protease-related functions, as well as on pathway modules spanning metabolism, inflammation, endocrine signaling, and cancer circuitry. Docking against the ten hub proteins yielded binding energies from −6.00 to −11.49 kcal/mol, with the strongest predicted interaction observed for MMP9 (6ESM, −11.49 kcal/mol), followed by TNF (1GVS, −10.28 kcal/mol) and ESR1 (4XI3, −9.63 kcal/mol). For MMP9, daidzin showed a more favorable docking score than B9Z, the co-crystallized reference ligand of 6ESM (−10.54 kcal/mol), and sirtinol, a general breast cancer-related comparator (−10.59 kcal/mol), supporting the selection of 6ESM for dynamic validation. In 200 ns simulations, both complexes exhibited global stability by RMSD, whereas daidzin-6ESM maintained higher hydrogen bond counts, most frequently 3−5 with transient increases, together with a SASA profile showing reduced phase-like variation relative to sirtinol-6ESM, consistent with more persistent interfacial organization. MMGBSA further supported stronger binding for daidzin-6ESM, with ΔTOTAL of −46.86 ± 3.83 kcal/mol compared with −14.12 ± 8.99 kcal/mol for sirtinol-6ESM, driven by stronger van der Waals and electrostatic contributions despite a larger polar solvation penalty. ADMET predictions indicated higher aqueous solubility for daidzin (−2.784 log mol/L) than sirtinol (−4.120 log mol/L) but lower predicted permeability and intestinal absorption (0.240 log Papp and 59.319%) than sirtinol (0.934 log Papp and 92.147%). Thus, the predicted ADMET profile indicates a safety-exposure trade-off, with favorable safety-related flags for daidzin, including AMES negativity and absence of predicted hepatotoxicity, but possible absorption-related limitations. DFT analysis indicated a larger frontier gap for daidzin (ΔE 10.6214 eV) than sirtinol (ΔE 9.3543 eV), higher hardness (η 5.3107 versus 4.6772 eV), lower softness (σ 0.1883 versus 0.2138 eV^-1^), and a modestly higher electrophilicity index (ω 1.0894 versus 1.0463 eV), consistent with greater electronic stability for daidzin. Collectively, these computational findings prioritize the daidzin-MMP9 interaction as a mechanistically plausible and hypothesis-generating route for further investigation in breast cancer, rather than demonstrating direct MMP9 inhibition or therapeutic efficacy. If subsequent preclinical testing demonstrates reproducible activity with an acceptable safety margin, advancement toward clinical investigation may be warranted. Overall, the present findings provide a mechanistic and methodological foundation for focused experimental validation and future therapeutic development. Thus, the present study should be interpreted as a computational prioritization framework that supports experimental testing of the daidzin-MMP9 hypothesis, not as definitive evidence of anti-breast cancer activity.

## Supporting information

S1 Table Docking grid center coordinates used for molecular docking. (DOCX)

S2 Table The interactions between daidzin and target genes. (DOCX)

S1 Fig Modelling interaction diagram of daidzin with target genes. (DOCX)

## Abbreviations

ADMET: Absorption, Distribution, Metabolism, Excretion, and Toxicity
BBB: Blood-brain barrier
CNS: Central nervous system
DFT: Density functional theory
GO: Gene Ontology
KEGG: Kyoto Encyclopedia of Genes and Genomes
MD: Molecular dynamics
MMP: Matrix metalloproteinase
MMGBSA: Molecular Mechanics/Generalized Born Surface Area
PDB: Protein Data Bank
PPI: Protein-protein interaction
Rg: Radius of gyration
RMSD: Root-mean-square deviation
RMSF: Root-mean-square fluctuation
SASA: Solvent-accessible surface area
