# Supplementary material for: Mechanistic insights into daidzin from Glycine max against breast cancer via network pharmacology and multi-level molecular modeling
Source: PLoS One. 2026 Aug 11;21(8):e0355672. doi: 10.1371/journal.pone.0355672 (PMC13460629; doi:10.1371/journal.pone.0355672)
Supplement: Supplementary file 1 [file pone.0355672.s001.docx]

**S1 Table. Docking grid center coordinates used for molecular docking.**

| **Target genes** | **Target**  **PDB ID** | **Grid center x** | **Grid center y** | **Grid center z** | **Grid size** | **Spacing** |
| --- | --- | --- | --- | --- | --- | --- |
| ALB | 6YG9 | 41.848 | 4.685 | 25.295 | 60 × 60 × 60 | 0.375 Å |
| TNF | 1GVS | 21.806 | 8.469 | 23.661 | 60 × 60 × 60 | 0.375 Å |
| MMP9 | 6ESM | 4.258 | 47.690 | 16.264 | 60 × 60 × 60 | 0.375 Å |
| CASP3 | 3PD1 | 37.268 | 13.828 | 66.771 | 60 × 60 × 60 | 0.375 Å |
| SRC | 4F5B | -11.549 | 21.259 | -0.062 | 60 × 60 × 60 | 0.375 Å |
| ITGB1 | 3T9K | 32.935 | -19.428 | -19.858 | 60 × 60 × 60 | 0.375 Å |
| MMP2 | 1QIB | 68.879 | 33.351 | 19.523 | 60 × 60 × 60 | 0.375 Å |
| ESR1 | 4XI3 | -2.794 | -7.787 | 29.585 | 60 × 60 × 60 | 0.375 Å |
| IL2 | 4NEJ | 6.300 | 19.772 | 16.777 | 60 × 60 × 60 | 0.375 Å |
| HSP90AA1 | 2QG2 | -12.500 | 29.250 | 11.222 | 60 × 60 × 60 | 0.375 Å |
