# Supplementary material for: Mechanistic insights into daidzin from Glycine max against breast cancer via network pharmacology and multi-level molecular modeling
Source: PLoS One. 2026 Aug 11;21(8):e0355672. doi: 10.1371/journal.pone.0355672 (PMC13460629; doi:10.1371/journal.pone.0355672)
Supplement: Supplementary file 2 [file pone.0355672.s002.docx]

**S2 Table. The interactions between daidzin and target genes.**

| **Target genes** | **Target PDB ID** | **Binding energy (kcal/mol)** | **Hydrogen bonds** | **Van der Waals interaction** | **Hydrophobic interaction** |
| --- | --- | --- | --- | --- | --- |
| ALB | 6YG9 | -8.24 | Glu17, Lys162, Arg186 | Lys20, Ala21, Leu115, Glu131, Glu132, Lys136, Leu155, Lys159, Phe165 | Leu135, Tyr138, Leu139, Ile142, Ala158, Tyr161, Lys162 |
| CASP3 | 3PD1 | -7.46 | Arg64, Ser120, Gln161, Ser205, Arg207, Ser251, Asp253 | His121, Ala162, Tyr204, Phe252, Phe256 | Cys163, Trp206, Arg207 |
| SRC | 4F5B | -6.15 | Gln147, Lys155, Glu179 | Ala148, Glu149, Glu150, Tyr152, Gly154, Tyr187 | Phe153 |
| ITGB1 | 3T9K | -7.25 | Asn434, His453, Lys517, Asp597, Val598, Asn629 | Leu435, Phe454, Tyr485, Ala593, Gly595, Ala596, Asn599, Phe625 | His453, Leu592, Asp597 |
| MMP2 | 1QIB | -7.78 | Ala165, Ala167, Ala169, Pro221 | Leu163, Leu164, Glu202, His205, Ile222, Tyr223 | His166, Ala167, Phe168 |
| IL2 | 4NEJ | -6.54 | Ile24, Gly27, Asn30, Asn71, Gln74, Asp84, Leu85, Ile86 | Asn26, Glu67 | Met23, Ile24, Leu70, Leu85 |
| HSP90AA1 | 2QG2 | -6.00 | ASp71, Ser72, Asn155, Asp156, Gly177, Gly181 | Ser68, Lys69, Leu70, Gln159, Asp175, Thr176, Met180, Arg182 | Lys153, Glu178, Pro179 |
