# Supplementary material for: Mechanistic insights into daidzin from Glycine max against breast cancer via network pharmacology and multi-level molecular modeling
Source: PLoS One. 2026 Aug 11;21(8):e0355672. doi: 10.1371/journal.pone.0355672 (PMC13460629; doi:10.1371/journal.pone.0355672)
Supplement: Supplementary file 3 [file pone.0355672.s003.docx]

**S1 Fig. Modelling interaction diagram of daidzin with target genes. (A) Daidzin-6YG9, (B) Daidzin-3PD1, (C) Daidzin-4F5B, (D) Daidzin-3T9K, (E) Daidzin-1QIB, (F) Daidzin-4NEJ, (G) Daidzin-2QG2, and (H) Daidzin-4XI3.**

**S1 Fig. *Continued.***

**S1 Fig. *Continued.***
